# Disease and “Broken Windows”

**DOI:** 10.1289/ehp.113-1281318

**Published:** 2005-10

**Authors:** Leo Meléndez

**Affiliations:** Public Health Seattle-King County Local Hazardous Waste Management Program, Seattle, Washington, E-mail: leo.melendez@metrokc.gov

Frumkin’s editorial in the May 2005 issue of *EHP* (Frumkin 2005) was very interesting and enlightening. On page A291, Frumkin cited several studies that endorse the “broken windows theory,” noting that

Part of this effect may well be due to the disorder and squalor of the environment. Poor people and people of color are disproportionately exposed to “broken windows.”

It is interesting that the “broken windows” are considered to cause disease and health inequity. What happened first: the “broken windows,” or the lack of social skills and the abandonment of the population who live in such places? As a scientist, I find it very difficult to accept that “broken windows” are associated with the number of cases of gonorrhea and are associated with causality. The cases of venereal diseases (VD) are more related to the social skills and social behaviors of the people living in the community. They also have a lack of respect for property, and destruction of property often occurs.

If we say the reverse is plausible, what would happen if we got a grant and fixed all of the “broken windows” in a particular community, with no other intervention, and observed the trend of VD? With the assumptions and inferences made in Frumkin’s editorial, this would have a positive effect in reducing cases of VD. My instincts tell me that this would not be the case. The “broken windows” are a consequence of the behaviors of that particular community and they are not the cause of the behaviors. The “broken windows” are what I consider “collateral damage” of people lacking the necessary social skills to overcome certain challenges, such as socioeconomic stress and the lack of maintenance provided by building owners. These people show their frustration and anger many times against property, as well as other people.
